# Renin Angiotensin System and COVID-19 Infection

**DOI:** 10.34172/apb.2022.001

**Published:** 2021-01-23

**Authors:** Francesco Ferrara, Antonio Vitiello

**Affiliations:** ^1^Pharmaceutical Department, Asl Napoli 3 Sud, Dell’amicizia street, Nola, Naples, Italy.; ^2^Pharmaceutical Department, Usl Umbria 1, XIV Settembre street, Perugia, Italy.

**Keywords:** ACE-2, RAS, COVID-19, Sars-CoV-2, Renin

## Abstract

***Purpose:*** The new coronavirus, called SARS-CoV-2, is responsible for the recent global pandemic COVID-19. The status of the global pandemic COVID-19 is currently underway, and the virus has caused about 1.11 million deaths. Several SARS-CoV-2 vaccines are in phase 3 clinical trials. Pending the availability of safe and effective vaccines, pharmacological treatments are experimental and aimed at avoiding the most serious complications of the infection.

***Methods:*** This article explores and describes the scientific evidence in the literature and the scientific pharmacological and molecular rationale to consider drugs that modulate the reninangiotensin system (RAS) system as therapeutic agents that if administered appropriately can help the host organism to fight SARS-CoV-2 infection.

***Results:*** It is known from the 2003 SARS epidemic that the critical receptor for SARS-CoV entry into host cells is the angiotensin-converting enzyme 2 (ACE2), the strain involved in the current SARS-CoV-2 epidemic is similar to the SARS-CoV strain involved in the 2002-2003 SARS epidemic. ACE-2 is part of the RAS system, the modulation of this enzyme could be of therapeutic efficacy.

***Conclusion:*** Depending on pharmacological knowledge, and epidemiological evidence in the literature based on current knowledge of the mechanism of penetration of SARS-CoV-2 in cells, and the role of ACE-2 in the inflammatory state of infection, therapeutic treatments that modulate RAS could be a weapon to fight COVID-19 infection.

## SARS-CoV-2

SARS-CoV-2 virus has spread rapidly in several countries, causing a global pandemic that to date has already caused more than 1 million deaths. SARS-CoV-2 is a family of RNA viruses that can infect humans and cause respiratory tract infections and respiratory distress syndrome. Studies have shown that SARS-CoV-2 has about 80% genome similar to the SARS-CoV responsible for the 2003 epidemic. Clinical experts and scientists have described SARS-CoV-2 infection in three phases, the first asymptomatic or slightly symptomatic, the second moderately severe characterized by a pulmonary inflammatory state, the third very severe phase characterized by a generalized inflammatory state affecting all tissues and not only the lungs.^1,2^ Biochemical interaction studies have shown that SARS-CoV-2 uses the angiotensin-converting enzyme 2 (ACE2) receptor protein to penetrate cells. ACE-2 is also a conversion enzyme that is part of the renin-angiotensin system (RAS) system. To date, there is as yet no conclusive evidence on the role of the RAS system and ACE-2 in the three stages of SARS-CoV-2 infection and, since modulations in the expression of ACE-2 and RAS in the various stages of COVID-19 infection have been shown, widely used drugs indicated for cardiovascular diseases such as ACE inhibitor (ACEi) or angiotensin II receptor blocker (ARB) may modulate this system directly or indirectly representing a potential positive or negative role in the evolution of the disease.^3-5^

## The role of ACE-2 and SARS-CoV-2

ACE-2 is a type I transmembrane metal-carboxypeptidase with homology to the ACE protein which is an enzyme known to be a key player in the RAS and a target of the ACEi family for the treatment of cardiovascular disease. The expression of ACE-2 has been shown primarily in vascular endothelial cells, renal tubular epithelium, Leydig cells in the testicles and lung epithelial cells. ACE-2 has been identified as a functional receptor of SARS-CoV and SARS-CoV-2 for entry into host cells and subsequent viral replication. Viral entry into respiratory tract cells is a critical step that causes direct lung injury. However, the role of ACE-2 in the pathophysiology of SARS-CoV-2 infection is much more complex than described, since ACE-2 is not only the receptor of virus entry, in fact the function of ACE-2 in infected lung tissue is currently not very clear, especially in the two most serious stages such as the second and third where it appears to have a protective role.^6,7^ These new discoveries could have a great impact on the development of effective therapies against SARS-CoV-2, or to make appropriate use of drugs already on the market that modulate the RAS system to better manage the infection. An interesting line of research to prevent cellular entry of the virus is that of anti-ACE-2 antibodies that could be used to block the binding of SARS-CoV-2 to the receptor, or a block upstream of the RAS cascade with direct renin inhibitors to decrease the expression of ACE-2.

## The renin-angiotensin system (RAS)

RAS is a complex cascade event that plays an important role in the control of hydrosaline homeostasis, blood pressure and plasma volume. The cascade begins with renin splitting angiotensinogen, a peptide produced in the liver, into an inactive decapeptide, angiotensin I (Ang I). The latter is converted to the active form by another proteolytic enzyme, the ACE-2 produced in the capillary endothelium where it converts angiotensin I (decapeptide) to angiotensin II (Ang II, octapeptide), a powerful vasoconstrictor. ACE, in addition to decoupling Ang I to Ang II, has another important function, it degrades bradykinin into inactive fragments. Bradykinin produces vasodilation through the production of prostaglandins and nitric oxide (NO) and inhibits the proliferation of smooth vascular muscle.

At the cellular level Ang II modulates cell contraction, cell growth, differentiation and apoptosis; it can promote the production of other cytokines, the expression of adhesion molecules and the subsequent recovery of inflammation cells, chemotaxis, macrophage activation. It has a proinflammatory action.^8-10^ The tissue increase of Ang II formation induces inflammation and Ang II is itself a powerful pro-inflammatory cytokine as well as a growth factor. Scientific evidence shows that Ang II activates the transcriptional factor NF-kb, the key factor of nuclear transcription in inflammatory and fibrotic diseases, and its activation allows the transcription of several inflammatory genes, including interleukin 6 and IL-1 which are responsible for the cytokinic cascade and the hyperactive inflammatory state that is generated especially in the third stage of infection.^11,12^

## Active agents on RAS and SARS-CoV-2

**Figure 1:**
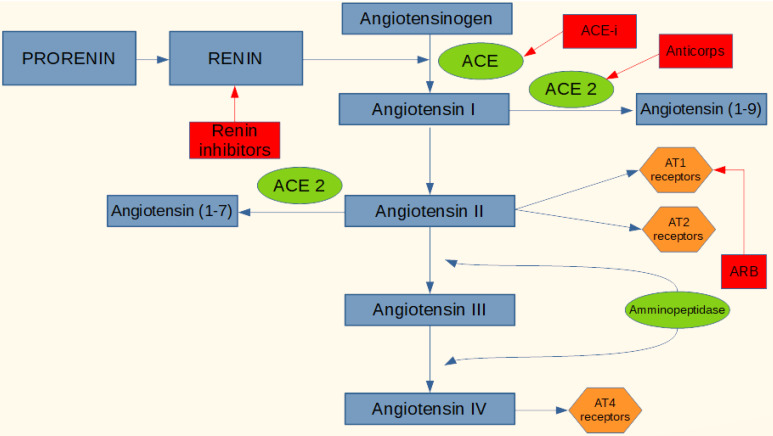
Active agents on the RAS system.

As previously reported SARS-CoV-2 uses the ACE2 as a receptor binding domain. Based on this, and considering the crucial role of the RAS system and the enzyme ACE-2 in the acute respiratory syndrome caused by SARS-CoV-2 and the correlation with the inflammatory status of lung tissues, it could be considered important to assume that the therapeutic agents acting on RAS, could modulate the viral infection with SARS-CoV-2, with an effect of improvement or worsening, and that managing these drugs in the most timely manner could be of great benefit. The active agents on RAS available may interfere with various stages of the system, an inhibitory action of the enzyme ACE (ACE-i), blocking Angiotensin II (ARB) receptors, inhibitors direct renin. ACE-i and ARB are the drugs of choice for the treatment of hypertension, heart failure and ischemic heart disease (Figure 1).

These drugs have an extraordinary therapeutic efficacy, but can also cause side effects such as hyperkalemia, hypotension, cough, angioedema. An increase in ACE-2 concentrations has already been documented both in murine models and in human patients treated with ACE-i and ARB. This is because by inhibiting the ACE pathway, angiotensin I is directed into the conversion pathway to angiotensin I-7, requiring a higher expression of ACE-2 to compensate for the greater amount to be converted. The same mechanism is with the use of ARB by blocking the Ang II receptor, in fact the system counter-regulates increasing ACE and ACE-2, in addition both ACE-i and ARB cause an increase in the concentration of renin and all the upstream mediators of the enzyme cascade, and this compensatory mechanism may be favorable or unfavorable depending on which stage of the SARS-CoV-2 infection the patient is. With the use of ACE-i or ARB and therefore an increase in the level of expression of the viral receptor mentioned above, an increase in infectious power and colonization could plausibly be obtained. In phase I of the infection, therefore, the virus is penetrating the cell and is replicating, perhaps in this phase it could be useful to administer a direct renin inhibitor which, acting upstream, lowers the concentrations of ACE and especially ACE-2, decreasing the concentration of receptor protein for the virus. On the contrary, in phase two or three of the disease, where there is an hyperactive inflammatory state and where it seems that ACE-2 may have a protective role in particular on the respiratory tract, it may be appropriate to increase ACE-2 with ACE-i or blocking the inflammatory effects of Ang II using ARBs, which also increase the expression of ACE-2 itself.^13-15^ In recent observational studies the most frequent comorbidities reported in patients with SARS-CoV-2 are often treated with ACE-i; however, the correlation between SARS-CoV-2 and treatment with ACE-i has not been evaluated and demonstrated in any of the studies. Based on epidemiological study, there is no evidence suggested that direct renin inhibitors cause an increase in ACE-2, this drug could therefore be used to manage the hypertension of patients which are in phase I of the infection, or in a preventive (non-virus positive patient) way to reduce the risk of contracting SARS-CoV-2 (Table 1).^16,17^

**Table 1:** Epidemiological evidence and related conclusions

**Conclusion**	**Epidemiological evidences**
ACE inhibitors and ARBs are associated with reduced risks of COVID-19 disease	Hippisley et al^18^ Risk of severe COVID-19 disease with ACE inhibitors and angiotensin receptor blockers: cohort study including 8.3 million people
No evidence that ACE inhibitors or ARBs affected the risk of COVID-19	Mancia et al^19^ Renin–Angiotensin–AldosteroneSystem Blockers and the Risk of Covid-19
RAAS inhibitors do not increase the risk of COVID-19 requiring admission to hospital, including fatal cases and those admitted to intensive care units, and should not be discontinued to prevent a severe case of COVID-19	Abajo et al^20^ Use of renin–angiotensin–aldosterone system inhibitors and risk of COVID-19 requiring admission to hospital: a case-population study
Chronic use of RAAS inhibitors does not negatively affect clinical course of COVID-19 in hypertensive patients	Felice et al^21^ Use of RAAS inhibitors and risk of clinical deterioration in COVID-19: results from an Italian cohort of 133 hypertensives.
There is no reason to modify current antihypertensive therapy	Gnavi et al^22^ Therapy with agents acting on the renin-angiotensin system and risk of SARS-CoV-2 infection

## Conclusion

In this article we suggest on the basis of today’s pharmacological and molecular knowledge and evidence literature, that by modulating the RAS system and the delicate ACE / ACE 2 Ang II balance in the best way and at the right times with common treatments such as ACE-i, ARB and direct renin inhibitors we can have important preventive and curative benefits to fight SARS-CoV-2.

## Ethical Issues

Not applicable.

## Conflict of Interest

The authors have no conflicts of interest to declare.
